# Re-exploration of the pharmacotherapeutic strategy for drug-induced liver injury: a narrative review

**DOI:** 10.3389/fmed.2026.1880114

**Published:** 2026-07-07

**Authors:** Yihui Liu, Yan Guo, Hong Xu, Ying Han, Hui Feng, Yutao Fang, Feng Pan

**Affiliations:** 1 Department of Gastroenterology, Hangzhou Red Cross Hospital, Hangzhou, China; 2 Department of Gastroenterology, Hangzhou Third Hospital, Hangzhou, China

**Keywords:** drug-induced liver injury, hepatoprotective drug, Hy’s, pharmacotherapy, therapeutic indication

## Abstract

The incidence of drug-induced liver injury (DILI) is on the rise, and discontinuation of suspected causative drugs is the core therapeutic principle. However, some patients may progress to moderate-to-severe liver injury, liver failure, or chronicity; thus, pharmacotherapy maybe considered a potentially valuable supportive treatment option in appropriate clinical contexts. At present, there is no unified consensus on the therapeutic indications and drug selection for DILI worldwide, and irrational practices such as prophylactic medication, indication-free medication and polypharmacy exist in China. This review proposes formulating therapeutic indications based on the severity, type of liver injury and Hy’s Law, identifying TBIL≥2 × ULN, cholestatic and mixed liver injury, acute liver failure (ALF), and high risk of ALF as indications for pharmacotherapy. For drug selection, N-acetylcysteine (NAC) is recommended by all domestic and international guidelines, Glucocorticoid (GC) may be used in selected cases, and hepatoprotective drugs such as magnesium isoglycyrrhizinate (MgIG) and bicyclol are widely prescribed in China. Pharmacotherapeutic regimens can be tailored according to the type and severity of liver injury.

## 1 Introduction

In recent years, the incidence of drug-induced liver injury (DILI) has been steadily rising, making it a serious public health concern. Current guidelines worldwide unanimously recommend withdrawal of suspected causative drugs as the first-line treatment for DILI. Most patients achieve spontaneous recovery after drug discontinuation. However, some develop moderate to severe liver injury, and a small number of cases even progress to liver failure or chronic liver injury (1–5). Accordingly, adjunctive supportive therapies including pharmacological therapy may have greater potential clinical value.

To date, domestic and international guidelines have not reached a unified consensus on the therapeutic indications and drug selection for DILI (1–5). Distinct from clinical practices in other regions, a wide variety of medications are used for DILI treatment in China, and inappropriate practices such as prophylactic medication and polypharmacy are prevalent (1). Therefore, it is urgent to formulate expert consensus and standardize the pharmacotherapeutic regimens for DILI. Based on the latest research advances, this article systematically elaborates on the pharmacotherapeutic strategies for DILI.

## 2 Withdrawal of suspected causative drugs cannot fully meet therapeutic needs for DILI

Guidelines worldwide explicitly recommend timely withdrawal of suspected causative drugs as the core intervention and fundamental therapeutic principle for DILI (1–5). Nevertheless, although liver function recovers spontaneously in most DILI patients after drug withdrawal, a small number of cases progress to liver failure and even chronicity. Studies have shown that 2 to 10% of DILI patients present with jaundice as the initial symptom (6); DILI accounts for approximately 20% of hospitalized patients with acute liver failure (ALF) (7). Meanwhile, DILI has become the leading cause of ALF globally with its proportion gradually increasing (8),and the overall mortality rate of drug-induced acute liver failure (DI-ALF) is about 10% (9, 10). In addition, the incidence of chronic DILI varies greatly due to factors such as different time definitions, with reported rates ranging from 3.4 to 39%, and patients with chronic DILI have a markedly elevated risk of liver cirrhosis (11, 12).

From the perspective of clinical practice, the diagnosis and management of DILI involve a variety of complex situations. In some patients, liver function parameters continue to rise rather than improve after withdrawal of suspected causative drugs. For liver injury induced by anti-tuberculosis and anti-tumor agents, clinicians need to carefully balance hepatoprotective therapy and treatment for the primary disease, given the imperative need to control the underlying illness. DILI patients with pre-existing liver diseases have a substantially higher mortality risk (13). Drug-induced autoimmune hepatitis (DI-AIH) requires targeted treatment with glucocorticoid (GC) (14–16). Current clinical guidelines also recommend GC for immune checkpoint inhibitor-associated liver injury (IALI) (17). Moreover, clinical research suggest that GC may be indicated for chronic DILI as well (18, 19). In addition, for drug-related vascular liver disorders such as hepatic sinusoidal obstruction syndrome (hepatic veno-occlusive disease), heparin or defibrotide are the preferred therapeutic options (20, 21).

Therefore, we consider that discontinuation of suspected causative drugs alone may not fully satisfy the therapeutic needs of patients with DILI. To date, robust evidence confirming that pharmacotherapy lowers the risk of liver failure, improves patient prognosis or halts progression to chronic liver injury remains lacking. Even so, pharmacotherapy may offer a relatively favorable cost-effectiveness profile when compared with other supportive strategies including artificial liver support and liver transplantation. For patients with malignancies, tuberculosis and other primary illnesses that require urgent treatment, pharmacological approaches may potentially facilitate the recovery of liver function. Individualized supportive care combined with pharmacotherapy may serve as a valuable component in the clinical management of DILI.

## 3 Irrational medication use in DILI pharmacotherapy

Although pharmacological interventions are warranted for DILI in selected cases, undue drug use and drug abuse are not recommended in clinical practice.

### 3.1 Prophylactic medication

Liver injury is among the most frequent adverse drug reactions in clinical practice. For medications with prominent hepatotoxic potential, such as anti-tuberculosis and anti-cancer agents, combined hepatoprotective drugs are widely used for prophylaxis to lower the risk of subsequent liver damage. Nevertheless, the value of this preventive strategy remains debatable in current research. Several studies have demonstrated that prophylactic hepatoprotective treatment can effectively reduce the incidence of DILI (22–27). Conversely, other reports indicate that such preventive medication not only fails to avert liver injury, but may even exacerbate the risk of hepatic damage and bring other potential hazards (28–30). In the absence of high-level evidence from clinical trials, the actual clinical benefits of prophylactic hepatoprotection remain uncertain. Accordingly, current domestic and international guidelines do not recommend routine prophylactic hepatoprotective therapy for all patients (1–5). Of note, prophylactic drug use is uncommon during administration of anti-infective agents, another major class of DILI-inducing drugs. For such high-risk populations, close monitoring combined with timely intervention may be more cost-effective than universal prophylactic intervention.

### 3.2 Non-indicated medication

Hepatoprotective drugs are frequently overprescribed for patients with mild DILI, namely those presenting only slightly elevated transaminases without jaundice, fatigue or other clinical symptoms. Given the liver’s adaptive capacity to drugs, close follow-up can be implemented to avoid unnecessary drug discontinuation. Alternatively, sole drug withdrawal combined with observation may be adopted to prevent disease progression and facilitate DILI diagnosis. Notably, blind early administration of hepatoprotective drugs may interfere with the diagnostic process. Our small-sample retrospective analysis further suggests that combined drug discontinuation and watchful waiting may improve the accuracy of DILI diagnosis (31).

### 3.3 Polypharmacy

Polypharmacy is commonly observed in the clinical management of DILI in China, where patients are often prescribed five to six hepatoprotective drugs concurrently (32–40). Nevertheless, current evidence fails to demonstrate superior therapeutic outcomes from the combined administration of two or more such drugs. A retrospective analysis revealed that short-term efficacy was not improved by regimens containing three or more hepatoprotective drugs compared with those using one or two drugs. Additionally, dual-drug combination showed no clinical advantage over monotherapy, while substantially raising medication costs (41). Accordingly, the concurrent use of two or more hepatoprotective drugs is not recommended for patients with DILI. The Chinese Guidelines for DILI does not recommend the combined use of two or more hepatoprotective drugs that primarily lower ALT. For mixed DILI, one ALT-lowering drug may be combined with another drug to alleviate cholestasis (1).

In conclusion, clinically unnecessary medications should be used with great caution in routine practice. Prophylactic medication ought to be individualized and administered prudently rather than adopted universally, and monotherapy is generally preferable. For most patients with DILI, treatment with one or two drugs appears adequate. Meanwhile, it remains unclear whether dual-drug regimens can satisfy the therapeutic needs of those with moderate to severe DILI and DI-ALF.

## 4 Indications of pharmacotherapy

Simply discontinuing suspected causative drugs cannot address all clinical scenarios of DILI. Irrational medication use remains prevalent in clinical settings, which underscores the pressing need to develop a unified consensus on the indications for pharmacological treatment of DILI.

Currently, major discrepancies and gaps exist in therapeutic recommendations across international DILI guidelines. The Asia-Pacific guidelines explicitly list coagulopathy, hepatic encephalopathy and liver failure as indications for hospital admission (4). European guidelines and the CIOMS consensus advise immediate intervention upon the onset of jaundice (2, 5). In comparison, the Chinese and ACG guidelines offer no detailed specifications or recommendations regarding treatment indications (1, 3). Despite this, pharmacotherapy is widely administered to DILI patients in clinical practice across China.

The absence of well-defined therapeutic indications has led to widespread clinical uncertainties: whether discontinuation of culprit drugs alone carries risks of disease progression or adverse outcomes without additional pharmacotherapy; how to determine the optimal timing for initiating medication, and distinguish cases requiring immediate treatment from those suitable for mere drug cessation and observation; and whether early pharmacological intervention can effectively lower the risks of liver failure and chronicity.

Accordingly, establishing a unified clinical consensus on the indications for DILI pharmacotherapy has emerged as a critical and urgent task.

Based on current research evidence, we propose that pharmacotherapeutic principles for DILI can be formulated according to the severity and type of liver injury.

*Severity of liver injury*: According to European guidelines and the CIOMS consensus, pharmacotherapy may be considered for grade 2 or higher liver injury (ALT ≥ 5 × ULN or ALP ≥ 2 × ULN with TBIL ≥ 2 × ULN, or symptomatic hepatitis) (2, 5).

*Type of liver injury*: Pharmacotherapy may be considered for patients with hepatocellular liver injury accompanied by jaundice. Current clinical studies indicates that cholestatic DILI is associated with a higher risk of chronicity and prolonged recovery (9, 42, 43). Accordingly, early pharmacological intervention may be considered for cholestatic and mixed-type DILI.

*Hy’s law*: It is defined as serum ALT or AST levels ≥3 × ULN together with TBIL ≥2 × ULN developing during medication. Its core diagnostic criteria are largely consistent with the definition of grade 2 liver injury. Large cohort studies have confirmed that around 10% of patients fulfilling Hy’s Law may progress to acute ALF (44–46). Currently, Hy’s Law is recommended by domestic and international DILI guidelines as a prognostic marker for this condition (1–5).

*Simplified treatment indications*: For clinical convenience, we propose simplifying the principles of DILI pharmacotherapy, and recommend initiating pharmacotherapy for patients meeting any of the following indications: ① TBIL ≥ 2 × ULN; ② Cholestatic and mixed liver injury; ③ ALF and high risk developing liver failure. Notably, these simplified treatment indications merely reflect our proposals based on available evidence and are not recognized by guidelines.

For patients who do not meet the above indications, clinicians shall perform individualized assessment by integrating clinical manifestations, dynamic changes in liver function markers and conditions of the primary disease, so as to determine the necessity of pharmacotherapy and prevent unindicated overtreatment Table 1.

**Table 1:** Indications for pharmacotherapy of DILI.

Classification	Core criteria	Evidence
Severity of liver injury	Grade 2 or higher liver injury: ALT≥5 × ULN, or ALP ≥ 2 × ULN accompanied by TBIL≥2 × ULN, or symptomatic hepatitis.	European guidelines and CIOMS consensus (2, 5).
Types of liver injury	Hepatocellular: *R* ≥ 5 and TBIL≥2 × ULN.Cholestatic: *R* ≤ 2.Mixed: 2 < *R* < 5.	European guidelines and CIOMS consensus (2, 5).Clinical studies reveal a higher risk of chronic disease and delayed recovery in these patients (9, 42, 43).
Hy’s law	ALT/AST ≥ 3 × ULN and TBIL≥2 × ULN.	1. Its core criteria are highly consistent with the definition of Grade 2 liver injury (5).2. Clinical studies confirm nearly 10% of criterion-qualified patients can develop ALF (44–46).3. Both domestic and international guidelines recommend it as a prognostic marker for DILI (1–5).
Simplified treatment indications	Fulfill any of the below criteria:1. TBIL≥2 × ULN.2. Cholestatic and mixed liver injury.3. ALF and high risk developing liver failure.	Based on relevant guidelines (2, 5) and clinical studies (9, 42–46)

## 5 Selection of pharmacotherapeutic regimens

Recommendations for pharmacological treatment of DILI differ across international guidelines. The ACG guidelines recommend N-acetylcysteine (NAC) for APAP-induced ALF and GC for DI-AIH, while offering no specific drug recommendations for other clinical scenarios (3). The Asia-Pacific and European guidelines share similar recommendations on NAC and GC. In addition, they advise targeted agents for liver injury induced by specific drugs including leflunomide and valproic acid (2–4). The CIOMS consensus explicitly recognizes the clinical value of NAC, UDCA and magnesium isoglycyrrhizinate (MgIG) in DILI management (5). The Chinese guidelines, meanwhile, give formal recommendations for NAC, MgIG and bicyclol (1).

### 5.1 NAC

NAC is the only specific antidote approved by the FDA for APAP-related hepatotoxicity (47). Multiple studies have demonstrated that NAC can markedly improve transplant-free survival in patients with non-APAP-induced ALF (48–53), though other investigations have yielded contradictory findings (54–56). Evidence also indicates that the efficacy of NAC for non-APAP-induced ALF varies substantially by etiology and cannot be generalized, with favorable outcomes limited to ALF triggered by certain non-APAP drugs (57). Currently, NAC is well recognized for the early management of adult DI-ALF. While several studies have reported that NAC ameliorates liver function parameters in patients with non-ALF DILI (58, 59), its overall clinical value and benefits in this setting remain to be further elucidated.

### 5.2 GC

GC is not a routine therapy for DILI. Their well-established indications are limited to drug reaction with DRESS syndrome, DI-AIH and IALI. Nevertheless, GC is commonly prescribed for moderate to severe DILI and DI-ALF in real-world clinical practice. Published studies have yielded conflicting results regarding the efficacy of GC: several investigations have demonstrated therapeutic benefits (60–64), whereas others have reported no favorable effects (65–67). While most of these studies are retrospective, one prospective randomized controlled trial (RCT) verified that GC accelerate recovery in patients with severe DILI (68). Given the paucity of high-quality evidence to either support or refute its clinical value (69), the use of GC in DILI remains controversial. Considering that GC can benefit specific patient populations and may reduce the need for artificial liver support or liver transplantation, we believe current indications for GC use fail to meet clinical needs. High-quality RCTs are urgently needed to clarify the efficacy, initiation timing and optimal dosage of GC, so as to standardize their clinical application in DILI patients. GC in relevant studies include methylprednisolone, prednisone, prednisolone and dexamethasone, which are administered orally or intravenously (1, 68).

### 5.3 Hepatoprotective drugs

Unlike practices in other countries, a broad spectrum of medications is applied to treat liver injury in China. Chinese guidelines divide these hepatoprotective drugs into two groups: those primarily lowering ALT and/or AST, and those mainly reducing ALP and/or GGT (1). Nevertheless, this classification fails to consider the mechanisms of action of hepatoprotective drugs, and its scientific rationale for DILI management remains questionable.

*MgIG*: It is the only drug officially indicated for acute DILI in China, and RCT have verified its therapeutic efficacy for this condition (70).

*Bicyclol*: As the first oral drug undergoing registration trials for acute DILI, it has been validated in RCT to effectively reduce serum ALT and AST levels in affected patients (71).

The CIOMS consensus and Chinese guidelines recognize the clinical value of MgIG for DILI (1, 5). Meanwhile, bicyclol receives strong recommendations in the DILI guidelines of China and Russia (1, 72). However, due to the lack of high quality evidence, such drugs are not recommended in other guidelines.

*Ursodeoxycholic acid (UDCA)*: Multiple clinical studies have demonstrated its efficacy for DILI (73–75), yet high-quality evidence remains insufficient. The CIOMS consensus and Chinese guidelines fully acknowledge the therapeutic role of UDCA in cholestatic DILI (1, 5), whereas international guidelines including ACG and EASL have not incorporated it into their recommended regimens.

*S-adenosylmethionine (SAMe)*: Multiple clinical studies have also validated its clinical benefits in the management of DILI (76–78). The Chinese guidelines recommend SAMe as a therapeutic option for cholestatic DILI, whereas no other clinical practice guidelines include relevant recommendations for this agent.

*Other hepatoprotective drugs*: The efficacy and safety of polyene phosphatidylcholine (PPC), glycyrrhizin (GL), tiopronin (TPN) and glutathione (GSH) for DILI have been validated in small-sample randomized controlled trials and real-world retrospective studies (79–84). These drugs are widely prescribed across clinical settings in China. However, given the scarcity of high-quality evidence, they are assigned only weak recommendations in the Chinese guidelines, and their use should be individualized according to clinical conditions. Of note, due to a paucity of high-quality evidence, international DILI guidelines do not recommend the aforementioned drugs Table 2.

**Table 2:** Classification of DILI pharmacotherapies.

Therapeutic drugs	Indications	Evidence
NAC	Specific antidote for APAP; DI-ALF	Domestic and international DILI guidelines (1–5)
GC	DRESS syndrome, DI-AIH, IALI; severe DILI and DI-ALF	Domestic and international DILI guidelines (1–5); clinical evidence (60–64, 68)
MgIG	Acute DILI	CIOMS consensus and Chinese DILI guidelines (1, 5); package insert indications
Bicyclol	Acute DILI	Chinese and Russian DILI guidelines (1, 72)
UDCA	Cholestatic DILI	CIOMS consensus and Chinese DILI guidelines (1, 5)
SAMe	Cholestatic DILI	Chinese DILI guidelines (1)
Other hepatoprotective drugs (PPC, CG, TPN, GSH)	Acute DILI	Chinese DILI guidelines (1)

### 5.4 Pharmacoeconomics

Prior investigations have constructed Markov models to perform cost-effectiveness analyses covering five hepatoprotective drugs: GL, PPC, MgIG, diammonium glycyrrhizinate, and GSH. Analysis results showed that MgIG achieved superior therapeutic efficacy but entailed higher treatment costs, with an incremental cost-effectiveness ratio (ICER) of 3500.57 per quality-adjusted life year (QALY) (85). Other pharmacoeconomic assessments relying on decision-analytic models reported that bicyclol tablets generated an ICER of 244.87723 and exhibited a more cost-efficient profile relative to tiopronin enteric-coated tablets and silybin capsules (86). One further research applied a decision-analytic framework to compare the pharmacoeconomic performance of four regimens, namely bicyclol tablets, tiopronin enteric-coated tablets, diammonium glycyrrhizinate injection, and GSH injection. The bicyclol group obtained the best overall efficacy score (4.5118), the lowest cost-effectiveness ratio (CER = 86.2667) and the smallest ICER (245.0118), accompanied by satisfactory safety outcomes. In comparison, the tiopronin enteric-coated tablet group yielded relatively inferior efficacy (4.1352) while carrying the minimum total treatment expenditure (296.9536). Taking the markedly effective rate as the primary endpoint, bicyclol showed the highest response rate (73.10%), as well as the lowest CER (5.32) and ICER (4.93) across all treatment cohorts (87). Another study established a decision-tree model for supplementary cost-effectiveness analysis (CEA). The CEA grounded on therapeutic efficacy scores demonstrated that bicyclol produced the most prominent clinical benefit (4.63562) and the lowest ICER (206.03270), whereas silybin reached the minimal CER (68.59987). When the complete liver function normalization rate was set as the outcome metric, bicyclol remained the dominant alternative, with the highest normalization proportion (83.562%), the lowest CER (4.63627), and the smallest ICER (4.63504). Follow-up sensitivity analyses further confirmed the robustness of these analytical outcomes (88).

### 5.5 Combined pharmacotherapy strategies

Based on current clinical practice, these combined regimens might be applicable to liver injury with different classifications and severity grades.

#### 5.5.1 By liver injury type

*Hepatocellular*: MgIG and/or bicyclol.

*Cholestatic*: UDCA and/ or SAMe.

*Mixed*: MgIG /bicyclol and UDCA/SAMe.

#### 5.5.2 By liver injury severity

*Mild liver injury*: PPC/GL/TPN/GSH.

*Moderate to severe liver injury*: MgIG/bicyclol and UDCA/SAMe.

*DI-ALF*: NAC and MgIG/bicyclol and UDCA/SAMe and GC Table 3.

**Table 3:** Combined pharmacotherapy.

Classification		Therapeutic drugs	Evidence
Types of liver injury	Hepatocellular	MgIG and/or bicyclol	Chinese DILI guidelines (1)
	Cholestatic	UDCA and/or SAMe	Chinese DILI guidelines (1)
	Mixed	MgIG /bicyclol and UDCA/SAMe	Chinese DILI guidelines (1)
Degree of liver injury	Mild liver injury	PPC/GL/TPN/GSH	Chinese DILI guidelines (1)
	Moderate to severe liver injury	MgIG/bicyclol and UDCA/SAMe	Chinese DILI guidelines (1)
	ALF	NAC and MgIG/bicyclol and UDCA/SAMe and GC	Domestic and International Clinical Guidelines (1–5); clinical research (60–64, 68)

It should be noted that domestic and international clinical guidelines have reached a general consensus on the use of NAC and GC for treatment. However, notable discrepancies exist regarding other therapeutic drugs, which are rarely discussed in current guidelines. This is primarily attributed to the scarcity of high-quality clinical evidence for these drugs. Large-sample, prospective randomized controlled trials are therefore urgently needed to verify their efficacy. Such studies will generate evidence to guide pharmacotherapy for DILI and facilitate the standardization of its clinical application.

### 5.6 Treatment selection for DILI based on pathogenetic classification

DILI is categorized into intrinsic, idiosyncratic and indirect types according to its pathogenesis.

#### 5.6.1 Intrinsic

NAC is the sole antidote approved by the FDA for intrinsic DILI induced by APAP (1–5). There is no specific antidote for intrinsic DILI unrelated to APAP. Withdrawal of suspected causative drugs is the primary intervention.

#### 5.6.2 Idiosyncratic

GC are indicated for immune-mediated idiosyncratic DILI complicated by hypersensitivity or autoimmune manifestations, including drug reaction with eosinophilia and DRESS syndrome and DI-ALH (1–5). For other types of idiosyncratic liver injury, no specific treatment drugs have been developed so far.

#### 5.6.3 Indirect

Current guidelines recommend timely initiation of GC therapy for IALI (1). For indirect DILI induced by other drugs, the use of GC remains controversial, and the efficacy of hepatoprotective drugs remains unclear.

Beyond these specific etiologies, current domestic and international guidelines provide no definitive medication recommendations for DILI categorized by pathogenic mechanisms. In routine clinical practice in China, treatment drugs are generally selected based on the type and severity of liver injury.

### 5.7 Pharmacotherapy for DILI in special populations

#### 5.7.1 Pediatric

A clinical study enrolled 30 children with idiosyncratic DILI, more than half of whom received corticosteroid therapy (89). NAC has been validated to treat pediatric APAP-related liver injury effectively (90, 91). Nevertheless, NAC administration is associated with a markedly reduced 1-year transplant-free survival rate in children with non-APAP-induced liver failure (92); accordingly, DILI guidelines from multiple countries do not recommend NAC for non-APAP-associated hepatic injury in children (1–3). Reduced GSH, SAMe, GL and UDCA are routinely applied as hepatoprotective drugs for pediatric DILI (93).

#### 5.7.2 Elderly population

Available studies suggest individualized treatment with ursodeoxycholic acid, NAC or corticosteroids for elderly patients with DILI as appropriate (94). Multiple domestic Chinese clinical trials have verified the definite efficacy and favorable safety of MgIG, UDCA, GSH and SAMe in elderly DILI management (95–97), whereas current relevant expert consensuses fail to provide standardized medication recommendations for older DILI patients (98).

#### 5.7.3 Pregnant women

Liver function spontaneously recovers in most pregnant patients with DILI after timely discontinuation of culprit medications (99–101), and short-term low-dose prednisone can accelerate hepatic recovery (102, 103). Case reports have documented the use of prednisone combined with ursodeoxycholic acid for gestational DI-ALF (104). NAC is indicated for pregnant women complicated with APAP intoxication (105); pharmacological evidence demonstrates that NAC crosses the placental barrier and neutralizes toxic metabolites within fetal hepatocytes, thereby efficiently alleviating secondary fetal liver injury (106). Except for the drugs mentioned above, the selection of hepatoprotective drugs for gestational DILI is mainly based on drug labeling. Only compound glycyrrhizin can be used discretionally after adequate benefit–risk assessment, while most other drugs are contraindicated during pregnancy.

Evidence on pharmacological treatments for DILI in special populations remains limited. High-quality clinical data are insufficient, and no unified consensus has been reached worldwide.

## 6 Prospects for pharmacotherapy of DILI

### 6.1 Promote high-quality clinical research

Most drugs used for DILI treatment lack robust clinical evidence. Well-designed multicenter RCTs are therefore warranted to define the optimal indications and dosage regimens of these drugs. The findings will support guideline revision and clinical decision-making. In addition, future pharmacotherapeutic research should prioritize high-risk conditions including DI-ALF and chronic DILI.

### 6.2 Establish unified therapeutic indications

Currently, no universal consensus has been reached on the pharmacotherapeutic indications for DILI. We should synthesize available research evidence, reconcile discrepancies in recommendations between domestic and international guidelines, and develop standardized therapeutic consensus with high clinical applicability. These efforts will also help standardize clinical medication practices, including reducing prophylactic drug administration and unnecessary polypharmacy.

### 6.3 Drug withdrawal: core of DILI treatment

The primary therapeutic principle for DILI is the timely withdrawal of suspected causative drugs. Pharmacological intervention is not the first-line option in most cases. When drug withdrawal alone fails to achieve the desired therapeutic effect, adjuvant pharmacotherapy is required.

## 7 Limitation

Several limitations of this review should be acknowledged. First, this study constitutes a narrative review rather than a systematic review or meta-analysis, which may introduce potential selection bias in literature inclusion and interpretation; the evidence synthesis presented herein reflects the authors’ perspectives and clinical experience, and thus may not be fully representative of the entire body of available evidence in this field. Second, the quality of clinical evidence supporting the majority of hepatoprotective drugs remains suboptimal: while MgIG and bicyclol have been validated in RCTs, most other hepatoprotective drugs are only supported by small-sample clinical studies or retrospective analyses, and high-quality, multicenter, large-scale RCTs remain lacking for most pharmacotherapeutic regimens discussed. Third, considerable geographic heterogeneity exists in the clinical practice of DILI pharmacotherapy, as many hepatoprotective drugs widely used in China (such as MgIG and bicyclol) are not commonly prescribed in Western countries; consequently, the treatment recommendations proposed in this review may have limited generalizability to regions with distinct healthcare systems and medication practices. Finally, the scientific validity and clinical rationale underlying the pharmacotherapeutic indications and drug selection strategies for DILI proposed in this review warrant further validation and support from high-quality evidence-based clinical research.
